# Parents' experiences of parenting a child with profound intellectual and multiple disabilities in France: A qualitative study

**DOI:** 10.1111/hex.13910

**Published:** 2023-11-06

**Authors:** Marie‐Anastasie Aim, Marie‐Christine Rousseau, Ilyes Hamouda, Any Beltran Anzola, Thierry Billette de Villemeur, Mathieu Milh, Kim Maincent, Katia Lind, Pascal Auquier, Karine Baumstarck, Lionel Dany

**Affiliations:** ^1^ LPS, Social Psychology Laboratory Aix‐Marseille University Aix‐en‐Provence France; ^2^ Department of Epidemiology and Health Economics AP‐HM Marseille France; ^3^ CEReSS, Research Centre on Health Services and Quality of Life Aix‐Marseille University Marseille France; ^4^ Polyhandicap Federation San Salvadour APHP Hospital Hyères France; ^5^ Methodological Support Unit for Clinical, Epidemiological and Economic Research AP‐HM Marseille France; ^6^ Pediatric Neurology Department APHP, GHUEP, Armand Trousseau Hospital Paris France; ^7^ Department of Pediatric Neurology AP‐HM, Timone Marseille France; ^8^ Committee for Studies Education and Care for People with Polyhandicap (CESAP) Paris France; ^9^ Alice Blum‐Ribes Pediatric Space Establishment of the Unions for the Management of Health Insurance Fund Establishments (UGECAM) Ile de France Group Montreuil France; ^10^ Department of Medical Oncology AP‐HM, Timone Marseille France

**Keywords:** care trajectory, parent, parenting work, polyhandicap, qualitative method, severe profound intellectual and multiple disabilities

## Abstract

**Introduction:**

Parents of persons with profound intellectual and multiple disabilities (PIMD) play a major and often lifelong role in the care and support of their child. A better understanding of parents' perspectives regarding their experiences of parenting their child with PIMD is essential to support them more effectively. Although this topic has been explored extensively in Anglo‐Saxon and Northern European countries, little is known about the experience of these parents in a highly institutionalized context such as that in France.

**Objective:**

We explored parents' experiences of the activities they performed to care for their child with PIMD (namely, the ‘parenting work’) in the French context.

**Method:**

Qualitative semistructured interviews were conducted by telephone with 34 parents of persons with PIMD aged 8–35. The resulting data were analyzed using thematic analysis.

**Results:**

The analysis highlighted the diversity of activities performed by parents as well as the influence of context on the forms of this parenting work. Five themes were developed: (1) navigating the challenges of obtaining medical recognition; (2) negotiating a concealed domain and becoming an expert; (3) unfolding medical and medicosocial care management; (4) navigating the challenges of daily living and (5) shaping one's child's possibilities.

**Conclusion:**

This study offers a better understanding of the challenges, levers and expectations of parents of children with PIMD in France. Contextual factors such as the lack of knowledge of PIMD among health professionals, access to knowledge and know‐how associated with care management, the administrative complexity of access to care and equipment, institutional issues (e.g., professional turnover) and societal ableism (e.g., access to infrastructures, interpersonal discrimination) shape the work parents perform to support their child's needs. It is necessary to consider contextual aspects to better support these parents and their children. Suggestions for applications are provided in the discussion.

**Patient or Public Contribution:**

One of the researchers, a parent of a child with PIMD, supported the research design and provided feedback on the study's procedures and manuscript.

## 1 INTRODUCTION

Persons with profound intellectual and multiple disabilities (PIMD) live with a combination of profound disturbances of neuromotor, perceptive and cognitive efficiency.^1^ Expressions of PIMD are multiple, evolving and specific to each person (e.g., epilepsy, respiratory and feeding issues, paraverbal and nonverbal communication challenges). This study focuses on forms of PIMD with much more severe and higher motor and cognitive impairment due to damage having arisen earlier in development (i.e., before 3 years old).^1^, ^2^ This evolving situation places these people in a position of high physical, psychological and social vulnerability and results in extreme dependency.^3^

Parents of persons with PIMD play a major and often lifelong role in their care and support regardless of the child's residence (i.e., parents' home or professional care setting). Studies on parental roles in the care management of their child with complex needs have highlighted issues such as performing medical procedures and managing (life‐threatening) emergencies, making life‐and‐death medical decisions, navigating interactions and relationships with health care and care system providers, advocating/fighting for services and sacrificing themselves for the caregiving role.^4^, ^5^, ^6^

Most of these studies have been conducted in Anglo‐Saxon and Northern European countries.^6^, ^7^, ^8^, ^9^ However, sociocultural, historical and structural (i.e., health care system) factors contribute to shaping experiences related to disabilities.^10^, ^11^ Therefore, the exploration of these experiences needs to be localized. Little is known about the complexity and dynamics of the challenges faced by parents throughout the journey of parenting their child with PIMD in France. In contrast to the extensive deinstitutionalization processes of other European countries, the French system relies on institutional settings that aim to offer graduated responses adapted to the severity of the person's health condition (rehabilitation centres, residential facilities [RFs], home care).^12^ However, these options are not necessarily adequate and may lack fluidity (e.g., lack of proper general care coordination, imperfect geographic coverage, lack of space in adult care facilities).^12^, ^13^, ^14^ A better understanding of parents' experiences of parenting their child with PIMD in this context is necessary to support them more effectively.^15^

Research in the field of disability and the autism spectrum has highlighted the relevance of conceptualizing parents' experiences of providing support for their child's needs in terms of trajectories and work.^16^, ^17^ The concept of trajectory includes not only the ‘physiological unfolding of a patient's disease but the total organization of work done [by patients, kin, and professionals] over the course of illness and the impact on those involved with that work and its organization’^18^,p.66 to control and cope with the consequences (e.g., physical, psychological, social, economic) of the illness situation. While this approach is mainly used in the field of chronic illnesses (e.g., cancer),^19^, ^20^ it can be relevantly transferred to the field of disability (e.g., chronic situation, impact on life, impact on social integration, ‘management’ that goes beyond the medical field, uncertainty regarding long‐term evolution).^16^ Relying on trajectories of care^21^ to consider the parental experience of parenting a child with PIMD enables us to consider parenting work performed inside and outside the medical environment as well as the contexts (sociostructural, relational, temporal) within which parenting work is performed. Drawing on the work of Singh,^17^ parenting work refers to the range of activities carried out by parents of a child with a disability to manage the disability over time in both public and private spaces. By conceptualizing parental care as work, we can recognize the importance, specialization and range of this work in shaping the PIMD trajectories of care.^17^ Furthermore, by considering contexts, we can avoid reinforcing the idea that the challenges encountered are linked to the pathology or syndrome itself rather than to a disabling context.^10^

To respect the sociocultural specificity of the French context, a terminological clarification must be made. Underlying epistemological and political issues regarding the conception of disabilities,^22^ the wording used within each language, and the specific cultural context all generate particular sense‐making and practices regarding disabilities. In some European and non‐European countries (e.g., Belgium, Switzerland, Italy and Canada as well as some African French‐speaking countries), such as France, PIMD is addressed by parents, professionals and public policies under the term ‘situation of polyhandicap’.^1^, ^23^ While the term ‘handicap’ is considered discriminatory in the English language, the French language does not have a specific term to distinguish between ‘handicap’, ‘disability’ and ‘impairment’. To speak of a ‘situation of polyhandicap’ rather than ‘polyhandicap’ reflects a desire to emphasize the context rather than the health condition, which is a way of reflecting a social approach of disability.^22^ We retained the use of ‘situation of polyhandicap’ in the original design and procedure of this study because it conveys a social sense for parents.

There is limited knowledge of the experience of parenting children with PIMD developed in the early stages of life in the French context. Using a qualitative approach to deepen the understanding of these parents' experiences, this study aimed to explore the forms of parenting work carried out to manage PIMD while highlighting the supports and challenges encountered, thereby identifying ways of supporting these parents more effectively.

## 2 METHODS

### 2.1 Study design

To contribute to ‘social justice’,^24^ we relied on qualitative interviews to give parents a voice. Qualitative semistructured interviews were chosen as the type most suited for parents to talk in depth about their experiences of caring for their child with PIMD.^6^ To benefit from an ‘insider’ perspective, we approached a parent with whom the research team had previously worked for feedback on the study design and procedure.

### 2.2 Participant recruitment

Criterion‐based purposive sampling^25^ was used to identify parents caring for children with PIMD. Eligible participants were over 18 years old, parents of persons with PIMD aged 8–35, capable of French fluency reasonable enough to participate in an interview, and willing to participate after being informed of the objectives and procedures of the study. We aimed to recruit a diverse sample to generate a broad understanding of parents' experience of caring for their children with PIMD. Because the French system relies on institutional settings, recruitment was conducted through a partnership with the three main care management modalities offered to persons with PIMD: specialized rehabilitation centres (*n* = 2; high level of medical and paramedical physical rehabilitation), RFs (*n* = 2; high level of psychosocial education and lower level of medical care) and specialized paediatric/neurological departments (*n* = 2, departments of two university hospitals; persons with PIMD living at least most of the week with their parents). The contact information of eligible parents was obtained via the care management modalities, and the research team mailed them a notice and a consent form by registered letter. After the parents received the documents, the first author (a psychologist and PhD in social psychology) contacted the potential participants to obtain their oral consent, answer their questions and set up an appointment for the research interview. Participation was voluntary, and each parent had the opportunity to participate or refuse without justification.

### 2.3 Participants

A total of 33 interviews were conducted with parents (26 with mothers, 6 with fathers and 1 with a couple) of younger children (*n* = 12; 8–10 years old), adolescents (*n* = 11; 11–20 years old) and adults (*n* = 10; 21–35 years old) with PIMD. The participants' characteristics are described in Table 1. The majority of participants were 50 years or older (55.9%) and currently unemployed (41.2%) or partially employed (32.4%). More than half of the participants lived with the other parent of their child (52.9%), nine (26.5%) lived with a new life partner and seven (20.6%) were single. At the time of the interview, 27 (79%) participants had at least partial knowledge of the aetiology of PIMD. Six participants moved from French overseas territories and departments, Eastern Europe, North and West Africa and South America to continental France so that their child could benefit from more appropriate care.

**Table 1:** Participant characteristics (*n* = 34).

Characteristics	*N* (%)
Care facilities	
Specialized rehabilitation centres	11 (33.3%)
Home care	12 (36.4%)
Residential facilities	10 (30.3%)
Age of persons with PIMD (years)	
8–10	12 (36.4%)
11–20	11(33.3%)
21–35	10 (30.3%)
Sex of persons with PIMD	
Male	17 (52%)
Female	16 (48%)
Parents' age	
35 years or less	4 (11.8%)
36–49 years	11 (32.3%)
50 years or more	19 (55.9%)
Parents' gender	
Masculine	7 (20.6%)
Feminine	27 (79.4%)
Marital status	
In a relationship (with the other parent)	18 (52.9%)
Single	9 (26.5%)
In a relationship (with a new partner)	7 (20.6)
Professional situation	
Full‐time job	8 (23.5%)
Reduced/variable time job	11 (32.4%)
Sick leave/disability leave	2 (5.9%)
Not willing/able to work	10 (29.4%)
Looking for a reduced‐time job	2 (5.9%)
Retired	1 (2.9%)

Abbreviation: PIMD, profound intellectual and multiple disabilities.

### 2.4 Data collection

A semistructured interview schedule was developed by the first and last authors (who were trained in qualitative methods and experienced in exploring lived experiences related to chronic and severe health situations). The interviews explored the participants' experiences with trajectories of PIMD (e.g., ‘Could you tell me the “story” about your child's situation of polyhandicap from the moment you became aware of her/his disability until today?’), their experiences related to PIMD (e.g., ‘Is there a word or words that would sum up what you have experienced in relation to your child's situation of polyhandicap?’), repercussions and adaptations related to the situation of PIMD (‘In the course of the “story” you shared with me, did you face any difficulties? Which ones?’), their experiences regarding care and support for their child (e.g., ‘Do you have any expectations regarding her/his care? Have these expectations changed over time?’) and their perspectives on the future (‘More broadly, how do you see the future? Do you have any plans, wishes or goals for your child?’). The questioning remained flexible to facilitate a full probing of relevant issues raised by the participants. The interviews were conducted by telephone^26^ in a confidential space by a single interviewer (the first author). One interview was conducted online using the video‐conferencing application Zoom (Zoom Video Communications Inc., version 5.0.3) at the request of the participant. The interviews, led by the first author, took place between October 2020 and October 2021. They were audio‐recorded digitally and transcribed verbatim, and all identifiers were removed. The interviews lasted between 41 and 260 min (mean = 112 min; SD = 46 min).

### 2.5 Data analysis

The interview transcripts were analyzed using thematic analysis.^27^ This method was chosen due to its flexibility,^28^ which allows the use of a constructionist perspective (i.e., individual experiences are constructed through social processes rather than fixed qualities ‘inside’ of them) and focuses on how various contexts (sociocultural, structural, relational, temporal) shape the participants' experience of caring for their child with PIMD. The analysis was conducted following Braun and Clarke's^27^ six phases of thematic analysis (i.e., familiarization with the data, coding, searching for themes, reviewing themes, defining and naming themes, writing up). We followed the 15‐point checklist for a good thematic analysis.^27^ Specifically, the first author immersed herself in the data and began data coding and preliminary theme generation concurrently with the interview fieldwork. These phases were iterative and responsive to new data and the identification codes and themes. As each interview was completed, it was handed over to the last author (who was trained in qualitative methods and familiar with the research field), who also immersed himself in the data. While the analysis was mainly led by the first author, the first and last authors frequently collaborated during the first three phases of the analysis. During the analysis, members of our pluridisciplinary research team (social psychology, public health, physical medicine and rehabilitation) regularly met to discuss what stood out in the data, which proved to be helpful for theme review. In addition to the recursive process of the analysis, a literature review enabled us to refine our themes in light of concepts such as parenting work and trajectories of care. Both team members and one parent provided feedback on the definition of themes and the writing of the manuscript, which led to discussion and some refinements. Overarching themes were identified, such as parents' experience of managing PIMD, which is discussed in this paper.

## 3 RESULTS

### 3.1 Themes identified

Five themes were developed from the data that illustrated parents' experiences of forms of parenting work that contributed to parenting their child with PIMD in the French context: (1) navigating the challenges of obtaining medical recognition, (2) negotiating a concealed domain and becoming an expert, (3) unfolding medical and medicosocial care management, (4) navigating the challenges of daily living and (5) shaping possibilities. Excerpts from the interviews are provided to demonstrate the interpretative adequacy of the analysis. The participants were assigned an identifier number to maintain both their own and their child's anonymity.

#### 3.1.1 Theme 1: Navigating the challenges of obtaining medical recognition

A common, dominant theme in the data was the challenges faced by parents in obtaining medical recognition for their child's atypical development and/or for a diagnosis/aetiology. The first manifestations of PIMD as well as the ‘entrance’ into the trajectories of PIMD varied according to the timing and type of PIMD manifestation experienced by each participant (e.g., pregnancy monitoring, physical appearance or health event at birth, atypical development, regression in acquisitions). Within these varied situations, the parenting work of medical recognition is entangled with the actions implemented by close relatives and healthcare professionals. While a few participants said that atypia was identified by professionals and that they were rapidly referred to the appropriate specialist or department, the majority reported a more complex journey. Approximately one‐third of participants mentioned that atypia was identified by the mother and, in one case, by a grandmother. A number of parents felt that their own or their partners' concerns were not properly heard or were downplayed by practitioners, particularly with regard to the mother's status, such as questioning parenting skills (e.g., the ability to properly breastfeed the baby, the ability to calm the baby) and dismissing mothers’ concerns as unfounded.

Every month we'd go to the pediatrician, and I'd talk to him and he would say ‘No, everything's fine from a neurological point of view, everything's fine, it's all ok’. […] It was really hard because he wasn't listening to me. (14, mother of an adolescent)

Parents who were not heard or properly guided expressed the need to take action to find new practitioners or services to meet their child's needs. The parenting work of obtaining medical recognition is subject to the need to manage uncertainties, multiple medical examinations and appointments with a variety of health professionals over a long period of time, which contributes to the physical and psychological exhaustion of parents.

They don't tell you, look ‘Your child has cancer […] um, he's got to have chemo’ where at least you could plan a bit, I understand, but [pause] all this undergoing tests, and they don't know, well, it all stays a bit mysterious and confuses everything. (33, mother of a younger child)

In addition to these challenges, participants emphasized the rarity and uncertainty of their child's health condition, which required specialized support, sometimes the need to ‘wait and see’, and medical advances, contributing to the challenge of obtaining medical recognition. Participants reflected on the meanings, aims, and feelings associated with obtaining medical recognition, such as the relief/understanding provided by the diagnosis, better adequacy of current and future care, understanding of how to prevent a similar health situation in future children or grandchildren and unwanted confrontations with certain information (e.g., life expectancy) or the value of a diagnosis in the context of a health situation for which only symptoms can be treated.

#### 3.1.2 Theme 2: Negotiating a concealed domain and becoming an expert

Another major theme involved the parenting work of discoveries and various negotiations related to the health system domain and the development of parental expertise in and through these discoveries and negotiations. The field of care and disability appears ‘concealed’ due to its invisibility at a societal level (i.e., unknown to people who are not concerned) and due to the complexity of access to related information (e.g., type and location of care, types of aid, administrative procedures, medical jargon). Entry into the world of care and disability, initially unknown by the vast majority of participants, led parents to familiarize themselves with medical and administrative jargon and the workings of the healthcare system to support their child's needs.

You begin to understand the, the system if you like, because it is a system, and when you find yourself in it, it becomes a system too. Because it's a system of money, a system of of knowledge, a system of, you have to come across the right person who can, who can guide you toward the right kind of equipment. (07, father of an adult)

The development of parents' expertise takes place in the context of a lack of support/guidance and information from professional actors/networks (e.g., health professionals, social workers) regarding the health conditions of the participants' children and the (types of) care, facilities and aid. This led parents to seek answers on their own (e.g., via exchanges between parents, magazines, associations, television programs, contact networks). In this context, parents who receive support from professionals (e.g., practitioners, social workers) emphasize the relief provided by such assistance.

In addition to an in‐depth understanding of their child, the expertise developed by parents also includes knowledge and skills related to medical and ‘technical’ care (e.g., administration of treatments, management of epileptic seizures and false routes, gastrostomy feeding, devices, carrying/transfers). The parents learned in various ways: by adjusting their practices and habits, by observing health professionals and, for some of them and on specific topics, through training within their child's care structure:

Well to start with, to set things up, I had to go there and learn all about things […] How to give personal hygiene… how to aspirate … NIV [non‐invasive ventilation] so when I went there on a Saturday morning to visit, I'd get there a bit earlier either when it was time for bathing and washing or … to, um, well, to practice doing it with them. (20, father of an adolescent)

#### 3.1.3 Theme 3: Unfolding medical and medicosocial care management

The third theme encompasses parenting work of medical and medicosocial care management through access to adapted care contexts, relationships with professionals and care organizations, and decision‐making about the child's care. While some parents were referred by professionals to appropriate facilities, a large number of participants mentioned that they had not been helped effectively or had independently identified professionals or facilities throughout the trajectory of care.

I thought that… regarding [the center], they would put me in touch with other doctors in other hospitals nearer to home […] they left me just like that, I mean I have to carry out all the necessary steps myself. (04, mother of an adult)

For the participants, the parenting work of managing medical and medicosocial care adapted to their child's needs and their own wishes involves complex institutional and health policy issues (e.g., geographical distribution of facilities, small number of facilities able to accommodate people with severe disabilities, number of places available, number of professionals in the facilities) that limit parental choices regarding the place or type of care as well as the number and type of activities offered by the facilities and generate waiting lists of up to several years.

In fact, you've got a kid who is four years on the waiting list… so he can, I don't know, see something else, be with kids just like him, get some care and… (01, mother of a younger child)

In this context, the search for facilities is experienced as worrying and time‐consuming, especially during the child‐to‐adult transition period.

Because the management of care also involves exchanges and coordination with professionals, participants mentioned complexity due to the unavailability of professionals, the frequency of turnover within the facilities, and the feeling of being unheard by professionals. A small number of participants emphasized the benefits of having encountered available and collaborative professionals. Contact with professionals (e.g., home and school carers, health professionals) who were unfamiliar with PIMD, its care specificities (e.g., pain management), and/or the uniqueness of each child (e.g., personal interests and tastes, communication style) led parents to highlight their own expertise and the benefits of sharing this expertise with professionals to meet their child's needs. Although a few parents, all of whom had a high level of education, took on this role of ‘educator’ or were able to co‐construct with the professionals, the majority did not feel they had the opportunity to do so.

When you go for one medical condition or another and there is in fact [PIMD], they will treat the condition he's in hospital for, but for all the others they aren't qualified. Most of the time […] It's quite difficult to really understand [PIMD]. (14, mother of an adolescent)

Furthermore, parents reported a complex decision‐making process regarding their child's devices (e.g., corset seat, gastrostomy) and potentially invasive and life‐threatening interventions.

#### 3.1.4 Theme 4: Navigating the challenges of daily living

A fourth major theme focuses on the parenting work of navigating the challenges of daily living, such as constant vigilance, adaptations and anticipations and the low recognition of day‐to‐day parenting work. For parents, care in everyday life (e.g., hygiene, feeding) is expressed by a constant presence and availability for their child and, more generally, by adaptations (e.g., home, transport, activities, social, family and professional life) and anticipations to meet their child's needs (‘I get everything ready before [my son] arrives […] See that everything is organized before my son arrives’, 02, mother of an adult).

These adaptations and anticipations are also linked to daily activities that aim to promote the accessibility of travel, places and activities. The parenting work of daily care is developed, in part, by confronting the lack of accessibility and the nonexistence of everyday adapted equipment for their growing child (e.g., nappies, bibs). Parents closely associate adaptations with the process of completing applications for aid, which is experienced as one of the most tiring aspects of parenting a child with PIMD. The timeframe for managing the applications and the mismatch between aid and needs led some parents to finance or build what they needed themselves.

All the same, you have lots of parents who muddle through or who set up a charity, who bake cakes so they can pay for a lift, so they can pay for some, the easy‐to‐use wheelchair or equipment, a mobility vehicle. (08, father of an adolescent)

Considering the plurality of parenting activities and the associated burden in the context of the scarcity of places in residential care, the timetables of day‐care facilities, the need to be available (e.g., follow‐up, unpredictable state of health), and the impossibility of leaving one's child alone or the complexity of entrusting the child to family and friends leads many parents to reduce, cease or rethink their professional activity and drastically restrict personal time, most parents felt a lack of recognition for their work and expressed the need for respite periods.

[The service providers] they take […] sorry 20 euros and 41 centimes, and sometimes they can have some left over, and me, for example, when I come along to take over, that's 75% of the lowest minimum wage […] that's 5 euros 91 […] what I mean is the system doesn't work for me. (17, mother of an adolescent)

#### 3.1.5 Theme 5: Shaping possibilities

This theme's content is shaped by the challenges encountered throughout the PIMD trajectories of care. It encompasses the range of parenting work that aims to go beyond the limits set by the medical and institutional fields and by sociocultural and political views on disabilities. Participants reflected on health professionals' statements about their child's abilities and capabilities (e.g., mobility, communication, access to a professional or affective life) due to PIMD and emphasized the value of an approach based on openness and recognition of their capabilities and possibilities rather than limitations and negation of their capabilities (‘Why does she say that my son won't have this or that, how does she know that my son, on the contrary, won't be able to do more?’, 17, mother of an adolescent). To promote their child's motor and communication capabilities, most parents researched and familiarized themselves with or even developed techniques, materials, devices and therapies that were not yet developed or were poorly developed in France.

I take my daughter abroad to get the therapy that exists there […] therapy that makes demands um on the child's motor, sensorial and cognitive skills, therapy that doesn't exist in France or isn't well known enough or perhaps not widespread enough. (12, mother of a younger child)

They also stressed the need to compensate for the shortage of professionals within facilities (e.g., physiotherapists, speech therapists) due to the unattractiveness of positions offered within facilities by seeking out professionals in private practice. Several participants expressed the need to fight with facilities, sometimes for several years, or to get involved in associations so that their child could (continue to) benefit from support, devices or therapies. In addition to motor and communication capabilities, participants recounted the work they had done to promote their child's potential in other areas of life, such as social life, leisure activities, spiritual life, and access to beauty/aesthetics (e.g., perfume, hairdressing, nail care, hair removal, clothes), to go beyond the social and structural limits imposed on disabled persons.

Have you ever seen a shop that specializes in clothes for the disabled? […] where you have the right to be different. To go into shops, something stylish […] why cut [the hair of her daughter] because she's disabled it has to be cut, why? […] she has the right to be beautiful [laugh]. (19, mother of a younger child)

Through the parenting work performed throughout the trajectories of care, parents became familiar with the issues related to their child's disability and took a critical stance on the social and structural limits imposed on disabled persons.

Er, well, we want um um it's to be included! We want, we want the handicap to be played down—they're people just like anyone else, except they have certain difficulties […] it's just a question of not making life complicated for them, that's all! (25, mother of a younger child)

## 4 DISCUSSION

This study qualitatively explored parents' experiences regarding the scope of activities they performed to care for their child with PIMD in the French context. The analysis identified a large range of parenting work shaped in and through relational, sociostructural, and temporal contexts. By offering insights into the challenges faced by parents throughout PIMD trajectories of care, the analysis also stressed how these challenges regulate the forms of parenting work and outline parental expectations.

The first theme highlighted that the parenting work of obtaining medical recognition is slow and uncertain due to the rarity and various forms of PIMD. Access to medical recognition allows parents to meet the emotional challenges generated by the absence of a diagnosis and to project themselves in terms of future (grand)parenthood, access to health care, and coordination of care.^29^, ^30^, ^31^ Because PIMD is a syndromic entity that involves many possible etiologies as time passes (e.g., neurodevelopmental delay), a PIMD diagnosis may be a long‐term process over many months or years. However, echoing the experience of parents of children with autism,^17^, ^32^, ^33^ the time and parental resources invested in this process may be increased due to professionals' nonresponsiveness to parents' concerns, lack of familiarity with PIMD, and a lack of information/guidance compensated by parents' proactivity. It is important to facilitate the medical recognition process by acting on these barriers. Paediatricians' and family doctors' representations of parents, the professional–parent relationship and how it may shape their practices toward parents^34^ must be explored. In addition, professionals should be provided with information on specialized neuropediatric consultations and trained to provide support and information to parents.

The second theme reflected parents' higher need and expectation for more accessible information (health system functioning, aid, care settings and therapies, care practices) and more effective social support throughout the PIMD trajectories of care.^35^ Participants felt ‘compelled’ to become experts by learning, seeking information and developing social networks to understand and navigate the complex functioning of the health system, the various forms of settings and care, the specificities of everyday care and the associated technical acts. The support provided to parents was variable, and this variation was likely to be exacerbated by the absence of a well‐defined pathway of support in France.^33^ There is a need for tailored information on ongoing sources of support, referral to care organizations, access to professionals familiar with PIMD (including health follow‐ups ‘beyond’ PIMD, such as the dentist, ophthalmologist, or gynaecologist), and the development of local and national interventions to support parents. The relevance of therapeutic education among informal caregivers was stressed by the main French association of parents of persons with PIMD.^36^ However, these programs are still underdeveloped in France and focus mainly on care practices, emergency management and curative and preventive actions.^37^ Themes 2 and 3 underscored the value of adding to these programs information about human, technical/material and financial support systems as well as places and types of care in the French system. In an institutionalized context such as France, the living and care settings of persons with PIMD necessarily guide the form and feasibility of implementing such programs.^37^ They must be made accessible in form (e.g., location, price, language) and in content to parents who desire them regardless of where their child lives and how they are cared for. Echoing the parenting work of opening up possibilities (theme 5), it seems imperative to recognize the potential of people with PIMD by formalizing adapted therapeutic education programs.^36^, ^37^

Data on the parenting work of medical and medicosocial care management (i.e., theme 3) provided further evidence of parental expectations in the practitioner‒parent relationship. In a context where parents do not feel heard by practitioners^5^ despite their expertise,^3^ interactions with professionals unfamiliar with PIMD during the care trajectory lead parents to adopt, when given the opportunity, the additional parenting work of training these professionals to ensure that their child's needs are met. It is important that parental expertise is recognized and valued. However, in a context where PIMD is drastically unaddressed in the training of health professionals (e.g., general practice, paediatrics), it is important that this lack of knowledge is not laid at the feet of parents. In addition to the development of curricula for health professionals in training, information sheets (e.g., initial and specific clinical pictures linked to PIMD, care and support needs) developed by specialists and parents should be created and provided to professionals to improve the service offered to persons with PIMD and their parents and, more broadly, to improve their care trajectories.^38^ Participants also stressed the low availability of professionals and the frequent turnover within care organizations, which makes exchanges between parents and professionals more complex. Referrals and spaces for exchange should be offered, and parents' expertise and care work must be acknowledged and validated in the provider–parent‒child relationship and in their child's care and life projects.^5^, ^39^ The participants concomitantly adopted a structural reading of the difficulties associated with high turnover and a lack of professionals in many facilities. In a context‐based mainly on institutional functioning, recruitment and employment retention tensions in the understaffed health, social and medicosocial sectors^40^ have a major impact on the availability of health professionals. To compensate and limit the impact of these dysfunctions on their child, parents adopted proactive measures.

The particularities of the French system also shaped the experience of parenting work, aiming to navigate the challenges of daily life (i.e., theme 4). Adding to the issues previously highlighted (e.g., constant vigilance and availability, housing adaptation, accessibility, economic and support issues) regarding the extent of daily parenting work^4^, ^9^, ^35^, ^41^, ^42^, ^43^ as well as the need to acknowledge and revaluate it, our results showed that in France, access to adaptation, financial, technical and human support is closely linked to administrative demands that may be inadequate and often complex, which leads parents to proactively seek substitutes. Overall, our results illustrate how the lack of adequacy and fluidity of the French system (e.g., a limited number of and spaces in facilities, mismatch of support and needs)^13^, ^44^ may add to parenting work and concerns and lead parents to develop alternative responses. While there is a strong expectation from families for access to more flexible, evolutive and diversified care modalities (e.g., day or temporary respite care), the current levels of human, financial and technical support are insufficient.^12^

The results of theme 5 showed that although parents embraced the view of disability as a medical issue by seeking medical recognition and various forms of therapies, their unmet needs due to confrontation with relational and sociostructural barriers led them to develop resistance to social and societal oppression of their child and people with disabilities.^17^, ^45^ Parents discussed sociostructural barriers and challenged the limits placed on their children by emphasizing the importance of focusing on the children's potential and abilities rather than their limitations, providing them with opportunities, and advocating for their social and societal inclusion. It is crucial to hear their voices and to develop organizations that respect these families' expectations, which implies the need to develop community care and community services.

Overall, parents' experiences revealed how heavily the French system relies on parental resources (e.g., literacy, social network, time, socioeconomic). This situation emphasizes the need to consider the impact of sociostructural constraints on parents' care burden^46^ and to explore their relation to parents' quality of life. While parental expertise and work must be recognized and valued, they should not be used to support and legitimize a dysfunctional healthcare system.^5^

## 5 STRENGTHS AND LIMITATIONS

This study is the first to explore parents' experiences of parenting a child with PIMD in France. By taking parental actions ‘seriously’ through the concept of parenting work, this research highlighted plentiful and usually overlooked parental practices. Furthermore, the use of qualitative interviews allowed us to give parents a voice while providing insights into a wide range of parenting work, including the challenges they faced and their expectations throughout the trajectory of care.

Limitations of this study must be acknowledged. Participants were recruited on a voluntary basis from contact information provided by facilities. It is possible that parents who refused to participate were faced with different challenges, parenting work or associated experiences than those who volunteered. Furthermore, although the native language of some of our participants was not French and some had migrated to receive care for their child in metropolitan France, only parents with sufficient French language skills were interviewed. The specificity of these experiences needs to be explored further because they can affect access to support and the shape of parenting work.^47^ It is also important to note that although a large number of emotions could be expressed over the telephone (e.g., crying, sniffing, laughing, hesitations, intonations), facial and gestural expressions were missed. However, when the study began, COVID‐19 was not quite over. This period was particularly trying and distressing for these families. By alleviating parents' anxiety about COVID‐19, a dematerialized interview helped to make interviews more acceptable and to free up room for discussion. With regard to the choice of telephone over videoconferencing, the majority of parents preferred the telephone because it left them free to carry out other tasks (e.g., preparing meals, going to work) in their busy daily lives. Finally, although we offered both parents the opportunity to participate in this research, the majority of our participants were mothers. Fathers' experiences of parenting work should be explored in more depth.

## 6 CONCLUSION

This study explored parents' experiences of parenting their child with PIMD in the French context. The results highlighted that contextual factors (e.g., lack of knowledge of PIMD among health professionals, access to knowledge and know‐how associated with care management, administrative complexity, institutional issues, societal ableism) shape the work parents perform to care for their children with PIMD. It is thus necessary to consider and deepen the understanding of how contexts shape parents’ ways of parenting their children with PIMD to avoid context‐generated burden and provide adapted support for parents and their children.

## AUTHOR CONTRIBUTIONS

All authors made a significant contribution to the work reported. Karine Baumstarck, Marie‐Christine Rousseau, Pascal Auquier and Lionel Dany contributed to funding acquisition. Karine Baumstarck, Marie‐Christine Rousseau, Lionel Dany and Marie‐Anastasie Aim contributed to research conceptualization. Karine Baumstarck and Any Beltran Anzola contributed to project administration. Thierry Billette de Villemeur, Mathieu Milh, Kim Maincent, Katia Lind, Marie‐Christine Rousseau, Any Beltran Anzola and Ilyes Hamouda provided participants' identification and access. Marie‐Anastasie Aim contributed to the collection of data. Marie‐Anastasie Aim and Lionel Dany contributed to the data analysis. Marie‐Anastasie Aim, Lionel Dany, Karine Baumstarck, Marie‐Christine Rousseau, Any Beltran Anzola and Ilyes Hamouda contributed to data interpretation. Marie‐Anastasie Aim, Lionel Dany, Karine Baumstarck, Marie‐Christine Rousseau, Any Beltran Anzola and Ilyes Hamouda contributed to drafting the manuscript and reviewing and editing the original draft.

## CONFLICT OF INTEREST STATEMENT

The authors declare no conflicts of interest.

## ETHICS STATEMENT

Ethical approval was granted by the Ile de France I Committee for the Protection of Persons (reference number: 20.03.20.54418). The participants provided oral and written informed consent.

## Data Availability

The data that support the findings of this study are available on request from the corresponding author. The data are not publicly available due to privacy or ethical restrictions.
